# Importance of PET/CT in Initial Workup of Head and Neck Squamous Cell Carcinoma

**DOI:** 10.1002/oto2.75

**Published:** 2023-09-20

**Authors:** Ryan N. Hellums, Priscilla F. A. Pichardo, Kenneth W. Altman, Ellen Penn, Kevin P. Stavrides, Nicholas C. Purdy

**Affiliations:** ^1^ Department of Otolaryngology–Head and Neck Surgery Facial Plastic Surgery Geisinger Medical Center Danville Pennsylvania USA; ^2^ Doctor of Medicine Program Geisinger Commonwealth School of Medicine Scranton Pennsylvania USA

**Keywords:** head and neck squamous cell carcinoma, PET/CT, second primary malignancies, staging

## Abstract

**Objective:**

Assess the impact of positron emission tomography/computed tomography (PET/CT) on disease staging at presentation in patients with head and neck squamous cell carcinoma.

**Study Design:**

Retrospective cross‐sectional review.

**Setting:**

Academic multicenter single institution (Geisinger Health System).

**Methods:**

All patients who had PET/CT imaging during workup for head and neck squamous cell carcinoma were included in the study. Pre‐ and post‐PET/CT clinical staging were recorded. Statistical analyses were performed for patients with a change in clinical staging or detection of second primary malignancies on PET/CT.

**Results:**

A total of 292 patients were included in the study, 238 of whom underwent PET/CT imaging as part of their initial workup. Twenty‐eight (11.9%) patients were clinically upstaged on PET/CT with 7 patients having treatment alterations based on imaging. Eighteen (7.6%) patients were found to have second primary malignancies on PET/CT.

**Conclusion:**

The current study further illustrates the importance of PET/CT in the workup of head and neck squamous cell carcinoma. Without the inclusion of PET/CT imaging, 19.3% of patients would have either been staged inappropriately or had second primary malignancies missed, again confirming the necessity of comprehensive functional imaging during the initial pretreatment workup.

Head and neck cancer accounts for nearly 4% of all malignancies diagnosed in the United States each year with approximately 15,000 deaths annually.^1^ Squamous cell carcinoma comprises more than 90% of all head and neck malignancies.^1^ As with all malignancies, optimal treatment planning and prognosis in head and neck squamous cell carcinoma (HNSCC) depends on accurate clinical staging.^2^ For example, while 5‐year survival rates are approximately 80% for isolated local disease, these survival rates drop to 50% and 20% with regional and distant metastatic disease, respectively.^3^ Similarly, the presence of lymph node metastasis in HNSCC decreases 5‐year survival rates by 40% to 50%, demonstrating the importance of accurate staging during initial cancer workup.^4^, ^5^

Imaging is essential to achieve accurate staging and is an integral part of the contemporary workup and initial staging for HNSCC. Conventional imaging techniques include computed tomography (CT) and magnetic resonance imaging for evaluation of the primary tumor and regional disease.^5^ Positron emission tomography (PET)/CT provides multiple advantages over traditional imaging modalities, specifically for the identification of regional and distant metastases, and secondary primary malignancies.^6^, ^7^ PET/CT has a high sensitivity and specificity for detecting distant metastatic disease, especially when compared to CT alone.^8^, ^9^ As such, PET/CT has become an effective imaging modality, and its importance has been well described in the HNSCC oncologic literature.

Not only is PET/CT invaluable for the initial staging and management of HNSCC, but it also plays an important role in the detection of second primary malignancies. Jones et al reported second primary malignancies in 9.1% of patients with HNSCC, with 14% of those being synchronous primaries.^10^ In a subsequent study, PET/CT detected occult second primary malignancies in 7.3% of patients, which were not identified on conventional workup and imaging.^11^ Kim et al demonstrated high sensitivity and specificity for PET/CT detection of second primary malignancies.^6^ In a reflection of the importance of PET/CT, current National Comprehensive Cancer Network (NCCN) guidelines recommend that all patients with locoregionally advanced disease undergo PET/CT as part of their initial workup.^12^

This study assesses the impact of PET/CT during initial workup and staging for patients with all stages and sites of HNSCC.

## Methods

The Office of Research, Compliance, and Institutional Review Board of Geisinger Medical Center approved this study. Included patients were diagnosed with HNSCC from December 1, 2019 to September 30, 2021 at a multisite tertiary care center. Patient data were collected and stored in the REDCap Head and Neck Cancer Patient Database.^13^, ^14^ Adult patients with a new diagnosis of HNSCC were included in this study. Exclusion criteria for the study were age <18 years, malignancies of unknown primary, recurrences, cutaneous malignancies, and patients with an incomplete workup or incomplete data within the electronic medical record. Patient demographics (age, sex, tobacco, and alcohol use), pretreatment workup, staging, treatment modalities, and time to treatment were recorded.

All imaging was reviewed by a head and neck neuroradiologist and multidisciplinary team during tumor board. The official radiologic assessment used for the purposes of this paper was that of the head and neck neuroradiologist. The cases were not blinded to the radiologist given that they were read in the tumor board setting. PET/CT imaging was performed in accordance with institutional guidelines. All patients were required to fast for at least 6 hours, follow a strict low‐carbohydrate diet for 24 hours, and avoid strenuous activity for 48 hours prior to imaging. Patients were required to have a fasting glucose level of less than 150 mg/dL. PET/CT was performed 60 minutes after the patient was injected with 7 to 17 mCi of intravenous fluorodeoxyglucose. Imaging included a full‐body noncontrasted CT with 5 mm slices followed by a full‐body PET scan.

Pre‐ and post‐PET/CT clinical staging were recorded separately. The staging was done in accordance with the American Joint Committee on Cancer 8th edition. The staging was established during the tumor board and subsequently confirmed by the investigative team during chart review. Patients whose staging changed after PET/CT were identified and recorded. Similarly, patients with biopsy‐proven second primary malignancies identified on PET/CT were recorded. All patients who were found to have a second primary malignancy were referred to the appropriate specialties for further evaluation and treatment of their disease after tumor board presentation. Early‐ and late‐stage disease was defined as stage I/II and stage III/IV, respectively. Time to treatment was defined as elapsed days from the date of initial otolaryngology consultation to the first day of treatment. Comparisons were performed using the *χ* ^2^ or Fisher's exact test, and 2‐sample Student's *T* test, as appropriate. A *P* value less than .05 was considered statistically significant.

## Results

**Figure 1:**
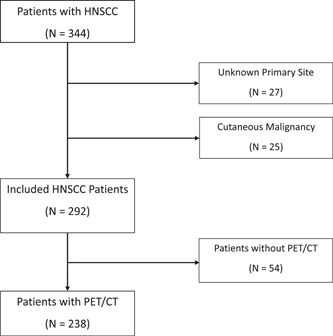
Study population. CT, computed tomography; HNSCC, head and neck squamous cell carcinoma; PET, positron emission tomography.

A total of 292 patients were included in the study, 238 of whom underwent PET/CT imaging as part of their initial workup (Figure 1). Table 1 demonstrates a similar demographic profile in each group. The most common primary site was the oropharynx (107 patients, 45%), followed by the oral cavity (59 patients, 24.8%), and the larynx (53 patients, 22.3%). The nasal cavity, nasopharynx, and salivary gland were less common with fewer than 10 patients each.

**Table 1:** Demographics

	PET/CT	No PET/CT	
Patients, N	238	54	
Age, mean	64.8	63.7	*P* = .504
Sex			
Male	188 (79%)	41 (76%)	*P* = .624
Female	50 (21%)	13 (24%)	*P* = .624
Tobacco use	178 (75%)	44 (81%)	*P* = .298
EtOH use	115 (48%)	28 (52%)	*P* = .638

Abbreviations: CT, computed tomography; PET, positron emission tomography.

PET/CT imaging findings changed the initial staging in 28 (11.8%) patients. This resulted in clinical upstaging in all cases. It is important to note that no patients were downstaged on PET/CT. Upstaging resulted from a change in primary site in 1 patient (3%), regional metastatic disease in 19 (68%) patients, and distant metastatic disease in 8 patients (29%). When stratified by early or late‐stage disease for all sites, the majority of upstaged patients had late‐stage disease (22 [13.8%] vs 6 [7.6%], *P* = .159). Table 2 demonstrates the change in staging based on the primary site and stage. Biopsy was performed for 16 patients with staging change on PET/CT, with a false positivity rate of 25%. These 4 patients, treated surgically for oral cavity disease, demonstrated no nodal disease identified on surgical pathology when PET/CT suggested either ipsilateral (1 patient) or bilateral (3 patients) nodal disease. Twelve patients did not have a biopsy to confirm PET/CT findings as the finding did not impact their treatment recommendation for chemoradiation. Seven (2.9%) patients had a change in management based on PET/CT findings. The extent of surgery changed in 5 patients, specifically bilateral neck dissections rather than ipsilateral. Two patients were treated with systemic therapy and radiation rather than surgery after identification of distant disease on PET/CT. Table 3 displays the primary site, staging, and treatment details for patients with altered treatment based on PET/CT results.

**Table 2:** Impact of PET/CT

Primary site	Staging impact		Second primary identified
	No change	Upstaged	
Hypopharynx	N = 7		
Stage I	‐	‐	‐
Stage II	‐	‐	‐
Stage III	‐	‐	‐
Stage IV	6 (85.7%)	1 (14.3%)	‐
Larynx	N = 53		
Stage I	5 (9.4%)	‐	1 (1.9%)
Stage II	2 (3.8%)	‐	‐
Stage III	17 (32.1%)	5 (9.4%)	2 (3.8%)
Stage IV	24 (45.3%)	‐	3 (5.7%)
Nasal cavity/nasopharynx	N = 10		
Stage I	‐	‐	‐
Stage II	2 (20%)	‐	‐
Stage III	1 (10%)	‐	‐
Stage IV	7 (70%)	‐	1 (10%)
Oral cavity	N = 59		
Stage I	11 (18.6%)	1 (1.7%)	1 (1.7%)
Stage II	5 (8.5%)	1 (1.7%)	‐
Stage III	4 (6.8%)	‐	1 (1.7%)
Stage IV	29 (49.2%)	8 (13.6%)	1 (1.7%)
Oropharynx	N = 107		
Stage I	32 (29.9%)	3 (2.8%)	3 (2.8%)
Stage II	15 (14%)	1 (0.9%)	‐
Stage III	28 (26.2%)	3 (2.8%)	2 (1.9%)
Stage IV	20 (18.7%)	5 (4.7%)	3 (2.8%)
Salivary glands	N = 2		
Stage I	‐	‐	‐
Stage II	1 (50%)	‐	‐
Stage III	‐	‐	‐
Stage IV	1 (50%)	‐	‐
Total	210	28	18

Percentages displayed are by primary site.

Abbreviations: CT, computed tomography; PET, positron emission tomography.

**Table 3:** Treatment Changes

Primary site	Staging (TNM)		Treatment	
	Pre‐PET/CT	Post‐PET/CT	Pre‐PET/CT	Post‐PET/CT
**Oral cavity**	T1N0M0	T2N2cM0	Partial glossectomy w/ipsilateral ND	Partial glossectomy w/bilateral ND
**Oral cavity**	T4aN1M0	T4aN2cM0	Composite resection w/ipsilateral ND	Composite resection w/bilateral ND
**Oral cavity**	T4bN1M0	T4bN2cM0	Composite resection w/ipsilateral ND	Composite resection w/bilateral ND
**Oral cavity**	T4aN0M0	T4aN2cM0	Composite resection w/ipsilateral ND	Composite resection w/bilateral ND
**Oral cavity**	T4aN0M0	T4aN1M1	Composite resection w/bilateral ND	Chemoradiation
**Oropharynx**	T1N1M0	T1N2M0	TORS radical tonsillectomy w/ipsilateral ND	TORS radical tonsillectomy w/bilateral ND
**Oropharynx**	T2N1M0	T4N0M0	TORS w/bilateral ND	Chemoradiation

Abbreviations: CT, computed tomography; ND, neck dissection; PET, positron emission tomography; TORS, transoral robotic surgery.

Broken down by primary site, clinical upstaging occurred in both early‐ and late‐stage oral cavity and oropharyngeal disease. Two (3.4%) patients with early‐stage oral cavity disease and 4 (3.7%) patients with early‐stage oropharyngeal disease were upstaged on PET/CT. Table 4 includes information for all early‐stage patients whose staging changed on PET/CT imaging. Interestingly, no patients with early‐stage laryngeal primaries were upstaged on PET/CT.

**Table 4:** Early‐Stage Patients With a Change in Staging on PET/CT

Primary site	Staging (TNM)		HPV (p16) (Y/N)	Treatment	
	Pre‐PET/CT	Post‐PET/CT		Pre‐PET/CT	Post‐PET/CT
**Oral cavity**	T1N0M0	T2N2cM0	‐	Partial glossectomy w/ipsilateral ND	Partial glossectomy w/bilateral ND
**Oral cavity**	T2N0M0	T2N1M0	‐	WLE w/ipsilateral ND	No change
**Oropharynx**	T4N1M0	T4N2M0	Y	Chemoradiation	No change
**Oropharynx**	T1N1M0	T1N2M0	Y	TORS radical tonsillectomy w/ipsilateral ND	TORS radical tonsillectomy w/bilateral ND
**Oropharynx**	T2N1M0	T2N2M0	Y	Chemoradiation	No change
**Oropharynx**	T2N1M0	T4N1M0	Y	Chemoradiation	No change

Abbreviations: CT, computed tomography; HPV, human papillomavirus; N, no; ND, neck dissection; PET, positron emission tomography; TORS, transoral robotic surgery; Y, yes.

Second primary malignancies were identified and confirmed with biopsy in 18 (7.6%) patients. Specifically, 5 (6.3%) patients with early‐stage and 13 (8.2%) patients with late‐stage disease were found to have second primary malignancies on PET/CT (*P* = .612). Second primary sites included 5 (27.8%) lung, 4 (22.2%) prostate, 3 (16.7%) cutaneous, 2 (11.1%) hematologic, 2 (11.1%) colon, 1 (5.6%) kidney, and 1 (5.6%) breast. Table 2 demonstrates which HNSCC patients were found to have second primary malignancies, broken down by primary site.

Of all patients in our study, 46 (19.3%) had a significant impact on prognosis following PET/CT imaging, meaning they were either upstaged or a second primary was identified. No patients were simultaneously upstaged and found to have a second primary malignancy. Clinical upstaging or second primary identification occurred more often in patients with late‐stage disease compared to early‐stage disease (35 [22%] vs 11 [13.9%], *P* = .137).

Time to treatment was similar for patients who underwent PET/CT during workup compared to those with CT imaging alone (43 vs 39 days, *P* = .17). Table 5 demonstrates the time to treatment from initial consultation stratified by treatment modality.

**Table 5:** Time to Treatment

Treatment	PET/CT	No PET/CT	
RT/chemo			
Patients, N	162	18	
Mean time to treatment, d	44.5	40	*P* = .210
Surgery			
Patients, N	74	35	
Mean time to treatment, d	41.5	37.5	*P* = .050

Abbreviations: CT, computed tomography; PET, positron emission tomography; RT, radiotherapy.

## Discussion

The current study demonstrates the importance of PET/CT imaging in the initial workup and staging for HNSCC. Results align with current NCCN guidelines for patients with advanced locoregional disease, but also suggest an important role for patients with early‐stage disease, with 13.9% of patients with stage I/II disease upstaged and 7.6% of patients found to have second primary malignancies on PET/CT. Not only does this study demonstrate the ability of PET/CT to prompt important changes in clinical staging, but it also redemonstrates the ability of PET/CT to detect second primary malignancies. Both of these properties have substantial prognostic implications for patients with HNSCC.

It is impossible to offer the treatment modalities with the highest cure rates and lowest possible morbidity without accurate staging. The clinical significance of changes in treatment based on initial staging imaging cannot be understated. Of all patients who underwent PET/CT for initial staging, 28 (11.8%) were clinically upstaged, with an associated change in treatment in 7 of these patients. Although the results of our study are limited by sample size, they are consistent with prior studies. Previous authors have suggested that PET/CT can change staging in up to 44% of patients with HNSCC, with treatment implications in up to 19% of patients.^11^, ^15^, ^16^ These treatment alterations suggest that PET/CT should be considered in all patients with HNSCC. While we do not assess clinical outcomes in our study, Ryu et al showed worse progression‐free and overall survival in patients who were upstaged on PET/CT when compared to patients with unchanged staging on PET/CT. While the treatment changes may seem minor when taken at face value, the results of Ryu et al study demonstrate the significance of correct initial staging and treatment.^15^

The importance of PET/CT in the detection of second primary malignancies was also demonstrated in this study. Approximately 8% of all patients were found to have second primary malignancies. Interestingly, 6.3% of all early‐stage HNSCC patients had second primary malignancies only discovered through PET/CT imaging. All early‐stage patients with second primary malignancies had oral cavity, oropharyngeal, or laryngeal primaries. Early‐stage HNSCC at these sites can arguably be staged without PET/CT, but in our population of patients that would have meant 5 patients could have had second primaries go unidentified during their initial staging. Interestingly, in a study by Jones et al, early locoregional disease had a higher propensity for second primary malignancy when compared to advanced locoregional disease.^10^ Although second primary malignancies such as lung, prostate, cutaneous, and so forth, should be discovered during traditional screening, PET/CT acts as an opportunistic screening modality in this population. Chen et al assessed the use of PET/CT as a screening modality in asymptomatic patients and discovered that ∼1.3% of the population were found to have malignancies.^17^ They also demonstrated high sensitivity and specificity for detecting malignant lesions.^17^ While PET/CT as a single screening agent for the general population is cost‐prohibitive, in a small cohort of HNSCC patients where imaging may alter their staging and treatment regimen or discover second primary malignancies, the use of PET/CT may be cost‐effective. Additional studies would be needed to determine the cost‐effectiveness of PET/CT in all stages of HNSCC.

A concern from prior authors in obtaining PET/CT routinely for all HNSCC patients is a delay in time to treatment initiation.^18^ However, the patients in our study did not have a significant delay in time to treatment when stratified by treatment modality, further supporting the use of PET/CT in the initial workup for HNSCC patients. This result is limited by the lack of uniformity in patients who did not undergo PET/CT. Multiple patients underwent CT chest, abdomen, and pelvis rather than PET/CT during their hospital admission, with some undergoing surgery that admission. These specific patients typically had prolonged hospital courses and waiting to perform PET/CT workup as an outpatient would have led to significant treatment delays. The other cohort of patients without PET/CT imaging were those with early‐stage malignancies that did not require PET/CT based on national guidelines. While our study does not show a significant delay in treatment initiation due to PET/CT, it is limited by its inability to assess the cost associated with PET/CT in this patient population. Prior studies have already demonstrated the cost‐effectiveness of PET/CT imaging in the posttreatment setting prior to adjuvant neck dissection for node‐positive HNSCC treated with chemoradiation.^19^ However, no studies have evaluated the cost‐effectiveness of PET/CT in initial staging for HNSCC. Current NCCN guidelines recommend PET/CT for advanced locoregional disease, which carries a high pretest probability for upstaging and inherently mitigates unnecessary costs. The cost‐effectiveness of PET/CT imaging for metastatic disease or second primary detection in early‐stage HNSCC disease is an important question. However, to adequately determine this a prospective study or thorough retrospective review would be required to evaluate life years gained, quality‐adjusted life years, incremental cost‐effectiveness, and incremental cost‐utility ratios. Additional studies are needed to adequately evaluate the cost‐effectiveness of PET/CT for all patients when considering effects on prognosis, outcomes, tumor persistence or recurrence, and treatment of second primaries at an earlier stage, all of which have major impacts on overall health care.

The additional cost of the workup for incidental findings on PET/CT and rates of false positivity is an interesting point of discussion that has been incompletely studied. In a Canadian study, Adams et al demonstrated that up to 75% of PET/CT scans had an incidental finding in the body of the radiologic report with 45% of those requiring follow‐up investigation.^20^ A cost analysis revealed an additional cost of ∼$130 per PET/CT for the recommended investigations, which included additional imaging modalities, endoscopic procedures, and biopsies. However, they did not assess costs associated with subspecialty consultations or delays in cancer treatment to further workup incidental findings, which can have significant cost implications. Shaban and Saleh investigated the rate of second primary malignancies on PET/CT imaging for patients with a known primary malignancy. They found that 4.67% of PET/CT scans had findings suggestive of a second primary, with a false positive rate of 31%.^21^ Given the potential delay in treatment for the primary lesion, additional studies comparing cost and time to treatment should be conducted. Clinicians must carefully balance the workup of incidental findings while simultaneously preparing the patient for treatment of their primary lesion.

An important limitation of this study is the lack of pathologic results for patients whose extent of surgery was altered based on PET/CT findings. It will be important to establish a positive predictive value for lymphadenopathy on PET/CT with regard to pathological nodal staging in early‐stage HNSCC. Prior studies have demonstrated a high negative predictive value in clinically N0 disease.^22^ The currently ongoing HN006 trial arm evaluating PET/CT and its histopathologic correlate for oral cavity SCC will provide valuable information for early‐stage disease. Until this concludes, PET/CT remains a reasonable consideration, specifically in early‐stage oral cavity SCC. Further studies are needed to assess the pathologic correlate for all primary sites to establish a positive predictive value for PET/CT assessment of nodal status.

## Conclusion

Our study illustrates the importance of PET/CT in the workup of HNSCC. Without the inclusion of PET/CT imaging, 19.3% of our patients would have been staged inappropriately or had a second primary malignancy missed. PET/CT is vital to accurate initial staging in HNSCC, which in turn is imperative for ideal treatment recommendations.

## Author Contributions

**Ryan N. Hellums**, design, conduct, analysis, presentation of the research, drafting of the manuscript; **Priscilla F. A. Pichardo**, design, conduct, and drafting of the manuscript; **Kenneth W. Altman**, design, conduct, revision, and approval of manuscript; **Ellen Penn**, design, conduct, and drafting of the manuscript; **Kevin P. Stavrides**, design, conduct, revision of manuscript; **Nicholas C. Purdy**, design, conduct, revision, and approval of the manuscript.

## Disclosures

### Competing interests

Ryan N. Hellums, Priscilla F. A. Pichardo, Ellen Penn, Kevin P. Stavrides, Nicholas C. Purdy: None. Kenneth W. Altman: Merck (consultant, speaker, publication advisory board [uncompensated]), Vindico (CME faculty in programs sponsored by Merck), AXDEV (Marketing consultant in a program sponsored by Merck), and Bayer (confidentiality agreement, uncompensated).

### Funding source

There were no sources of funding for this paper.
